# Peripheral blood immune cell subsets predict postoperative recurrence in colorectal cancer: a flow cytometry-based retrospective study

**DOI:** 10.3389/fonc.2026.1846793

**Published:** 2026-07-01

**Authors:** Yan Xu, Qing Cheng, Huilan Yang

**Affiliations:** Department of Clinical Laboratory, Suzhou Hospital of Integrated Traditional Chinese and Western Medicine, Suzhou, Jiangsu, China

**Keywords:** colorectal cancer, Cox regression, flow cytometry, Kaplan-Meier analysis, myeloid-derived suppressor cells, peripheral blood immune cells, postoperative recurrence, regulatory T cells

## Abstract

**Aim:**

To investigate preoperative peripheral blood immune cell subsets in colorectal cancer (CRC) and their predictive value for postoperative recurrence.

**Methods:**

A total of 158 patients with pathologically confirmed stage I-III colorectal cancer who underwent curative-intent surgical treatment were included. Fifty age- and sex-matched healthy volunteers were enrolled as controls for baseline immune cell reference values. Peripheral blood immune cell subsets were assessed preoperatively using flow cytometry. Clinicopathological and laboratory variables, including carcinoembryonic antigen (CEA), neutrophil-to-lymphocyte ratio (NLR), and albumin, were collected. RFS was defined as the time from surgery to postoperative recurrence or last follow-up. Kaplan-Meier analysis and Cox proportional hazards regression were used to evaluate factors associated with postoperative recurrence.

**Results:**

Among the 158 patients with stage I-III CRC, 58 developed recurrence during follow-up. Compared with healthy controls, patients with CRC had lower proportions of CD3^+^ T cells, CD4^+^ T cells, NK cells, and a lower CD4/CD8 ratio, but higher proportions of CD8^+^ T cells, Tregs, and myeloid-derived suppressor cells (MDSCs). Compared with the non-recurrence group, the recurrence group had a higher proportion of stage III disease, higher NLR, lower albumin, and higher levels of Tregs and MDSCs. Kaplan-Meier analysis showed poorer RFS in patients with stage III disease, NLR ≥3.5, high Treg levels, and high MDSC levels. In the combined multivariate Cox model, pathological TNM stage III, NLR ≥3.5, lower albumin, higher Treg levels, and higher MDSC levels were independently associated with postoperative recurrence. The combined model showed an apparent C-index of 0.77 and a bootstrap-corrected C-index of 0.75.

**Conclusion:**

Preoperative immune dysregulation is closely associated with CRC recurrence. A model incorporating immune subsets, inflammatory status, nutritional status, and pathological stage showed favorable discriminatory performance, suggesting that peripheral immune profiling may help refine recurrence risk stratification and personalized follow-up in CRC.

## 1 Introduction

Colorectal cancer (CRC) ranks among the malignancies with the highest incidence and mortality rates globally, and its disease burden continues to escalate (1, 2). Although curative-intent surgery combined with (neo)adjuvant therapies has significantly improved overall survival, postoperative recurrence and distant metastasis remain pivotal factors limiting long-term prognosis. Current risk assessment primarily relies on the tumor-node-metastasis (TNM) staging system, pathological risk factors, and certain serum biomarkers. However, considerable heterogeneity in recurrence risk exists among patients with the same stage, indicating that anatomical staging alone cannot fully capture tumor biology and host immune status.

Accumulating evidence underscores the critical role of the tumor immune microenvironment and systemic inflammatory responses in CRC progression, metastasis, and treatment response. The type, density, and spatial distribution of intratumoral immune cells can predict outcomes more accurately than conventional pathological staging (3). This insight has led to the international validation and clinical translation of the immunoscore for assessing recurrence risk in colon cancer (4, 5). Nevertheless, tissue-based immune assessment requires surgical or biopsy specimens and is constrained by factors such as sampling location, tumor heterogeneity, and assay costs. In contrast, the peripheral blood immune cell profile offers advantages of easy accessibility and repeatable monitoring, making it a promising complementary indicator reflecting the systemic immune ecosystem for preoperative risk stratification and follow-up management.

Among peripheral immune subsets, effector T cells and innate immune cells typically represent anti-tumor immune potential, whereas immunosuppressive cell populations are closely linked to immune evasion. Previous research has suggested that the proportion of regulatory T cells (Tregs) is elevated in the peripheral blood of CRC patients and possesses immunosuppressive functions (6). Standardized phenotypic definitions and gating strategies for Tregs have reached a consensus, providing a methodological foundation for cross-study comparisons (7). Furthermore, myeloid-derived suppressor cells (MDSCs) are considered potential prognostic markers and intervention targets, as they can promote CRC progression by inhibiting T-cell activation and fostering tumor-associated inflammation and metastasis (8). Natural killer (NK) cells play a vital role in tumor immunosurveillance, and reports of an association between reduced peripheral blood NK cell proportions and poor CRC prognosis are increasing (9). Concurrently, systemic inflammation and nutritional status are also closely related to CRC outcomes. Systematic reviews and meta-analyses have consistently concluded that an elevated pretreatment neutrophil-to-lymphocyte ratio (NLR) is associated with inferior survival (10), while hypoalbuminemia indicates an inflammation-nutrition imbalance linked to adverse preoperative outcomes and poor prognosis (11).

In particular, CD8^+^ cytotoxic T lymphocytes are major effector cells in antitumor immunity and can directly eliminate malignant cells through cytotoxic granule release and cytokine-mediated mechanisms. In CRC, a higher density of CD8^+^ T-cell infiltration has been associated with improved prognosis and constitutes a key component of the Immunoscore, highlighting the importance of cytotoxic T-cell-mediated immune surveillance. However, peripheral CD8^+^ T-cell proportions may not fully reflect their functional competence, as chronic antigen exposure and tumor-associated inflammation can lead to phenotypic dysfunction or exhaustion.

Based on this background, the present study aims to quantitatively characterize peripheral blood immune cell subsets in CRC patients using preoperative flow cytometry. By integrating these data with clinicopathological and laboratory parameters, we seek to investigate their association with postoperative recurrence risk and evaluate their predictive value. This could provide more actionable immunological evidence for individualized follow-up strategies and early risk intervention in CRC.

## 2 Materials and methods

### 2.1 Study design

This retrospective cohort study was conducted at a single tertiary care center (Department of Surgical Oncology). Patients with a confirmed pathological diagnosis of CRC who underwent surgical treatment between May 2023 and February 2025 were enrolled. Peripheral blood samples were collected from all patients preoperatively. The study protocol was approved by the Ethics Committee of Suzhou Hospital of Integrated Traditional Chinese and Western Medicine (Approval No. 2026-008) and conducted in accordance with the Declaration of Helsinki and relevant ethical guidelines. In addition to the 158 enrolled patients with CRC, 50 age- and sex-matched healthy volunteers (with no history of cancer) were recruited as a control group to establish baseline reference values for immune cells.

Inclusion criteria were: (1) age ≥ 18 years; (2) initial diagnosis of colorectal adenocarcinoma; (3) peripheral blood flow cytometry performed within 1 week before surgery; (4) underwent curative-intent resection.

Exclusion criteria included: (1) concurrent autoimmune disease; (2) hematologic malignancy; (3) preoperative neoadjuvant therapy; (4) active infection; (5) long-term immunosuppressive therapy.

### 2.2 Data collection

Demographic and general clinical data collected included age, sex, body mass index (BMI), smoking history (current/previous/never), comorbidities (diabetes, hypertension), Eastern Cooperative Oncology Group (ECOG) performance status, and American Society of Anesthesiologists (ASA) physical status classification. Age and BMI were recorded as continuous variables. Smoking history was determined based on medical record interviews. Comorbidities were identified based on documented prior diagnoses or history of long-term medication use. ECOG scores were recorded on a scale of 0–4 and stratified for statistical analysis. ASA classification was recorded as grades I-III.

Oncological parameters, derived from postoperative pathology and preoperative imaging, included tumor location (colon or rectum), maximum tumor diameter, differentiation grade (well/moderately differentiated vs. poorly/undifferentiated), lymphovascular invasion (LVI; positive or negative), and TNM stage. Pathological TNM staging was determined based on the final postoperative pathology report according to the 8th edition of the AJCC Cancer Staging Manual. Because this study focused on postoperative recurrence-free survival, stage IV patients were excluded from the primary recurrence and Cox regression analyses; therefore, TNM stage was stratified as stage I-II versus stage III. Postoperative adjuvant chemotherapy (yes/no) was determined based on discharge orders and oncology treatment records.

Laboratory parameters were obtained from the most recent test results within 1 week before surgery, including serum carcinoembryonic antigen (CEA), peripheral blood neutrophil count, lymphocyte count, and albumin (Alb) level. CEA was recorded as a continuous variable and stratified using the clinically common threshold of 5 ng/mL (12). NLR was calculated from the complete blood count and stratified using a predefined threshold of 3.5 (13). Alb was analyzed as a continuous variable.

Data on peripheral blood immune cell subsets were obtained from preoperative flow cytometry analyses and were uniformly expressed as percentages of lymphocytes (%). The measured parameters included CD3^+^ T cells, CD4^+^ T cells (CD3^+^CD4^+^), CD8^+^ T cells (CD3^+^CD8^+^), the CD4/CD8 ratio, Tregs (defined as CD4^+^CD25^high^CD127^low^), NK cells (CD3^-^CD56^+^), B cells (CD19^+^), and MDSCs (defined as HLA-DR^low^/-CD11b^+^CD33^+^). A standardized antibody panel and gating strategy were used for all samples to minimize detection bias.

All data in this study were extracted from the hospital’s electronic medical record system, laboratory information system, pathology system, and follow-up database. Two researchers independently collected and entered the data using a standardized data extraction form. Discrepancies were resolved by consensus with a third researcher. Peripheral blood collection and flow cytometry were completed within 1 week before surgery for all enrolled patients. Clinicopathological data were based on the final postoperative pathology report. Follow-up information was obtained through outpatient records, imaging data, and telephone interviews to ensure the accuracy and completeness of outcome data. No missing data were observed for the variables included in the final analysis; therefore, no imputation was performed.

### 2.3 Outcome definition

Recurrence was defined as local recurrence or distant metastasis confirmed by imaging and a clinical or multidisciplinary team discussion, or pathologically proven tumor recurrence during follow-up after curative-intent surgery. The follow-up period started from the date of surgery and ended on the date of the last follow-up or the date of confirmed recurrence.

### 2.4 Statistical analysis

All statistical analyses were performed using SPSS software (version 26.0, IBM Corp., Armonk, NY, USA) and R software (version 4.3.0). First, the normality of continuous variables was assessed using the Shapiro-Wilk test. Variables with a normal distribution were presented as mean ± standard deviation, and comparisons between two groups were made using the independent samples *t*-test. Non-normally distributed data were presented as median (interquartile range), and between-group comparisons were performed using the Mann-Whitney *U* test. Categorical variables were expressed as frequency (percentage), and comparisons were made using the *χ²* test or Fisher’s exact test. All tests were two-sided, and a *P*-value < 0.05 was considered statistically significant.

Recurrence-free survival (RFS) was defined as the time from surgery to the first documented postoperative recurrence. Patients without recurrence were censored at the date of last follow-up. Kaplan-Meier curves were generated to estimate RFS, and differences between groups were compared using the log-rank test.

Cox proportional hazards regression was used to evaluate factors associated with postoperative recurrence. Variables for multivariate Cox regression were selected using a predefined strategy that combined statistical screening with clinical relevance. Variables with P < 0.10 in univariate Cox regression were considered candidates for multivariate analysis. Clinical relevance was defined *a priori* as variables reflecting pathological disease burden, tumor marker status, systemic inflammation, nutritional status, or immune regulation. Based on these criteria, pathological TNM stage, CEA, NLR, albumin, Tregs, and MDSCs were included in the combined model. To reduce the risk of overfitting and multicollinearity, highly correlated variables were not entered simultaneously; therefore, pathological TNM stage was included as the representative staging variable, whereas pathological T and N stages were not included in the same model. Multicollinearity among variables included in the multivariate Cox models was assessed using the variance inflation factor (VIF), with VIF <5 considered to indicate no substantial multicollinearity. The proportional hazards assumption was evaluated using Schoenfeld residuals. Model discrimination was assessed using Harrell’s C-index, and internal validation was performed using bootstrap resampling with 1, 000 iterations to estimate optimism-corrected C-index values. All statistical tests were two-sided, and P < 0.05 was considered statistically significant.

## 3 Results

### 3.1 Baseline characteristics

A total of 158 patients with stage I-III CRC and 50 healthy controls were included in the analysis. There were no significant differences between the CRC and control groups in age, sex distribution, or BMI. Compared with healthy controls, patients with CRC had significantly higher preoperative CEA levels, higher NLR, and lower albumin levels.

Among the 158 patients with CRC, 58 patients developed recurrence during follow-up, whereas 100 patients remained recurrence-free or were censored without recurrence. Compared with the non-recurrence group, the recurrence group had a significantly higher proportion of stage III disease (77.59% vs. 32.00%, P < 0.001). The recurrence group also showed higher NLR levels and a higher proportion of patients with NLR ≥3.5. In contrast, albumin levels were significantly lower in the recurrence group than in the non-recurrence group. CEA level and the proportion of patients with CEA ≥5 ng/mL were numerically higher in the recurrence group, but the differences did not reach statistical significance. Other clinicopathological characteristics, including BMI, sex, smoking history, diabetes, hypertension, ECOG status, ASA classification, tumor location, differentiation, LVI status, and postoperative adjuvant chemotherapy, were comparable between the recurrence and non-recurrence groups (all *P* > 0.05; **Table 1**).

**Table 1:** Baseline characteristics.

Characteristic	Total CRC (n = 158)	Control (n = 50)	P	Non-recurrence (n = 100)	Recurrence (n = 58)	P
Age (years)	59.03 ± 10.46	56.88 ± 11.00	0.228	60.50 (53.00–65.00)	58.00 (50.00–67.75)	0.511
Male, n (%)	82 (51.90%)	27 (54.00%)	0.795	58 (58.00%)	25 (42.37%)	0.057
BMI (kg/m²)	23.67 ± 3.33	24.13 ± 2.48	0.295	24.00 ± 3.36	23.10 ± 3.22	0.097
Smoking history (current/previous), n (%)	48 (30.38%)	—	—	29 (29.00%)	19 (32.76%)	0.62
Diabetes, n (%)	34 (21.52%)	—	—	20 (20.00%)	14 (24.14%)	0.542
Hypertension, n (%)	48 (30.38%)	—	—	34 (34.00%)	14 (24.14%)	0.194
ECOG 0-1, n (%)	138 (87.34%)	—	—	89 (89.00%)	49 (84.48%)	0.41
ASA I-II, n (%)	143 (90.51%)	—	—	92 (92.00%)	51 (87.93%)	0.4
Rectal cancer, n (%)	98 (62.03%)	—	—	63 (63.00%)	35 (60.34%)	0.74
TNM stage III, n (%)	77 (48.73%)	—	—	32 (32.00%)	45 (77.59%)	<0.001
Poorly/undifferentiated, n (%)	41 (25.95%)	—	—	25 (25.00%)	16 (27.59%)	0.721
LVI-positive, n (%)	52 (32.91%)	—	—	34 (34.00%)	18 (31.03%)	0.702
Postoperative adjuvant chemotherapy, n (%)	108 (68.35%)	—	—	70 (70.00%)	38 (65.52%)	0.559
CEA (ng/mL)	4.53 (2.63–6.16)	2.20 (1.51–2.80)	<0.001	4.25 (2.62–6.00)	4.72 (2.67–6.36)	0.388
CEA ≥ 5 ng/mL, n (%)	63 (39.87%)	—	—	35 (35.00%)	28 (48.28%)	0.1
NLR	3.04 (2.02–4.41)	1.94 (1.43–2.26)	<0.001	2.82 (1.81–3.68)	3.84 (2.29–5.21)	0.004
NLR ≥ 3.5, n (%)	64 (40.51%)	—	—	32 (32.00%)	32 (55.17%)	0.004
Alb (g/L)	40.96 ± 4.40	43.09 ± 3.89	0.001	41.55 (39.18–44.52)	38.90 (36.38–42.63)	<0.001

CRC, colorectal cancer; BMI, body mass index; ECOG, Eastern Cooperative Oncology Group; ASA, American Society of Anesthesiologists; TNM, tumor-node-metastasis; LVI, lymphovascular invasion; CEA, carcinoembryonic antigen; NLR, neutrophil-to-lymphocyte ratio; Alb, albumin.

### 3.2 Comparison of peripheral blood immune profiles between CRC patients and healthy controls

Compared with healthy controls, patients with CRC showed a significantly altered immune cell distribution. Specifically, CRC patients had lower proportions of CD3^+^ T cells, CD4^+^ T cells, NK cells, and a lower CD4/CD8 ratio. In contrast, CRC patients had significantly higher proportions of CD8^+^ T cells, Tregs, and MDSCs. No significant difference was observed in B cells (CD19^+^) between the two groups (**Table 2**).

**Table 2:** Comparison of peripheral blood immune profiles between CRC patients and healthy controls.

Parameter	Control (n = 50)	CRC (n = 158)	*t/W*	*P*
B cells (CD19^+^, %)	10.55 ± 2.89	9.99 ± 3.26	1.138	0.258
CD3^+^ T cells (%)	69.83 ± 6.66	67.23 ± 8.29	2.257	0.026
CD4^+^ T cells (%)	32.54 ± 4.88	28.56 ± 4.88	5.023	<0.001
CD8^+^ T cells (%)	26.59 ± 5.09	31.39 ± 5.70	-5.648	<0.001
MDSCs (%)	1.92 ± 0.88	3.34 ± 1.51	-8.162	<0.001
NK cells (%)	15.51 ± 3.54	13.64 ± 4.12	3.131	0.002
Tregs (%)	4.23 ± 1.63	6.83 ± 2.52	-8.531	<0.001
CD4/CD8 ratio	1.27 (1.05-1.39)	0.91 (0.79–1.06)	6270.5	<0.001

CRC, colorectal cancer; MDSC, myeloid-derived suppressor cell; NK, natural killer; Treg, regulatory T cell.

### 3.3 Differences in immune subsets between patients with early and advanced CRC

Compared with patients with stage I-II CRC, those with stage III disease had significantly lower proportions of CD4^+^ T cells and NK cells, as well as a reduced CD4/CD8 ratio. Conversely, the proportions of CD8^+^ T cells, Tregs, and MDSCs were significantly higher in the stage III group. These findings suggest that stage III disease was associated with a more pronounced alteration of peripheral immune cell distribution (**Table 3**).

**Table 3:** Differences in immune subsets between patients with stage I-II and stage III CRC.

Parameter	Stage I–II (n = 81)	Stage III (n = 77)	*t/W*	*P*
CD4^+^ T cells (%)	30.15 ± 4.69	26.88 ± 4.54	4.446	<0.001
CD8^+^ T cells (%)	29.45 ± 5.61	33.43 ± 5.07	-4.681	<0.001
Tregs (%)	5.78 ± 2.03	7.93 ± 2.52	-5.879	<0.001
NK cells (%)	14.88 ± 4.08	12.34 ± 3.76	4.072	<0.001
MDSCs (%)	2.79 ± 1.39	3.91 ± 1.42	-5.010	<0.001
CD4/CD8 ratio	1.01 (0.90–1.17)	0.83 (0.70–0.93)	4813.500	<0.001

CRC, colorectal cancer; Treg, regulatory T cell; NK, natural killer; MDSC, myeloid-derived suppressor cell.

### 3.4 Comparison of preoperative immune subsets between recurrence and non-recurrence groups

Compared with the non-recurrence group, the recurrence group had higher MDSCs (3.90 ± 1.48% vs. 3.01 ± 1.43%, P < 0.001) and Tregs (8.00 ± 2.38% vs. 6.15 ± 2.34%, P < 0.001), and a lower CD4/CD8 ratio (0.87 [0.77–0.95] vs. 0.94 [0.79–1.13], P = 0.019). CD4^+^ T cells, CD8^+^ T cells, and NK cells did not differ significantly between groups (**Table 4**).

**Table 4:** Comparison of preoperative immune subsets between recurrence and non-recurrence groups.

Parameter	Non-recurrence (n = 100)	Recurrence (n = 58)	*t/W*	*P*
CD4^+^ T cells (%)	28.97 ± 4.92	27.84 ± 4.78	1.422	0.158
CD8^+^ T cells (%)	30.75 (27.17–34.92)	32.30 (28.92–35.32)	2498.000	0.148
MDSCs (%)	3.01 ± 1.43	3.90 ± 1.48	-3.686	<0.001
NK cells (%)	13.92 ± 4.11	13.16 ± 4.11	1.111	0.269
Tregs (%)	6.15 ± 2.34	8.00 ± 2.38	-4.741	<0.001
CD4/CD8 ratio	0.94 (0.79–1.13)	0.87 (0.77–0.95)	3551.500	0.019

MDSC, myeloid-derived suppressor cell; NK, natural killer; Treg, regulatory T cell.

### 3.5 Univariate and multivariate Cox regression analysis

Pathological TNM stage III was associated with an increased risk of recurrence compared with stage I–II disease (HR = 4.95, 95% CI: 2.65–9.23, P < 0.001). Elevated NLR (≥3.5) was also associated with higher recurrence risk (HR = 1.96, 95% CI: 1.17–3.29, P = 0.011), whereas higher albumin level was associated with a lower recurrence risk (HR = 0.90, 95% CI: 0.85–0.96, P < 0.001). Among immune cell subsets, higher Treg and MDSC levels were associated with increased recurrence risk (Tregs: HR = 1.28, 95% CI: 1.14–1.42, P < 0.001; MDSCs: HR = 1.34, 95% CI: 1.12–1.60, P = 0.001). CEA ≥5 ng/mL showed a borderline association with recurrence (HR = 1.55, 95% CI: 0.93–2.59, P = 0.096). Other clinicopathological variables and immune cell subsets were not significantly associated with recurrence in univariate analysis.

Variables with P < 0.10 in the univariate Cox regression analysis were entered into multivariate Cox proportional hazards regression models. Multicollinearity was assessed using the variance inflation factor (VIF), and all included variables had VIF values <5. In the clinical/laboratory model, pathological TNM stage III, NLR ≥3.5, and albumin remained significantly associated with postoperative recurrence. In the immune model, both Tregs and MDSCs were significantly associated with recurrence. In the combined model, pathological TNM stage III (HR = 2.90, 95% CI: 1.39–6.03, P = 0.004), NLR ≥3.5 (HR = 2.98, 95% CI: 1.69–5.25, P < 0.001), albumin (HR = 0.91, 95% CI: 0.85–0.97, P = 0.005), Tregs (HR = 1.18, 95% CI: 1.03–1.34, P = 0.017), and MDSCs (HR = 1.20, 95% CI: 1.00–1.44, P = 0.045) remained independently associated with postoperative recurrence. CEA ≥5 ng/mL was not significant in the combined model (**Tables 5**, **6**). Model discrimination was further assessed using Harrell’s C-index with 1, 000 bootstrap resamples for internal validation. The combined model showed the highest apparent C-index of 0.77 and a bootstrap-corrected C-index of 0.75 (**Supplementary Table 1**).

**Table 5:** Univariate Cox regression analysis for postoperative recurrence.

Predictor	*SE*	*Wald*	*P*	*HR* (*95% CI*)
Age, per year	0.013	0.42	0.515	0.99 (0.97–1.02)
Male sex	0.266	3.49	0.062	0.61 (0.36–1.02)
BMI, per kg/m²	0.038	2.21	0.137	0.95 (0.88–1.02)
Smoking history	0.281	0.44	0.509	1.20 (0.69–2.08)
Diabetes	0.311	2.62	0.106	1.66 (0.90–3.05)
Hypertension	0.310	0.99	0.321	0.74 (0.40–1.35)
ECOG 0–1	0.364	0.49	0.484	0.77 (0.38–1.58)
ASA I–II	0.406	0.47	0.492	0.76 (0.34–1.67)
Rectal cancer	0.269	0.03	0.854	0.95 (0.56–1.61)
Pathological TNM stage III vs I–II	0.318	25.23	<0.001	4.95 (2.65–9.23)
Poorly/undifferentiated tumor	0.294	1.12	0.290	1.37 (0.77–2.44)
LVI-positive	0.282	0.18	0.667	0.89 (0.51–1.54)
Postoperative adjuvant chemotherapy	0.280	0.91	0.340	0.77 (0.44–1.32)
CEA ≥5 ng/mL	0.261	2.77	0.096	1.55 (0.93–2.59)
NLR ≥3.5	0.264	6.48	0.011	1.96 (1.17–3.29)
Albumin, per g/L	0.031	11.11	<0.001	0.90 (0.85–0.96)
CD4^+^ T cells, per 1% increase	0.029	0.88	0.348	0.97 (0.92–1.03)
CD8^+^ T cells, per 1% increase	0.022	0.95	0.329	1.02 (0.98–1.07)
CD4/CD8 ratio	0.502	2.14	0.144	0.48 (0.18–1.29)
Tregs, per 1% increase	0.056	18.7	<0.001	1.28 (1.14–1.42)
MDSCs, per 1% increase	0.091	10.17	0.001	1.34 (1.12–1.60)
NK cells, per 1% increase	0.031	0.40	0.529	0.98 (0.92–1.04)

SE, standard error; HR, hazard ratio; CI, confidence interval; LVI, lymphovascular invasion; CEA, carcinoembryonic antigen; NLR, neutrophil-to-lymphocyte ratio; Treg, regulatory T cell; NK, natural killer; MDSC, myeloid-derived suppressor cell; Alb, albumin.

**Table 6:** Multivariate Cox regression analysis for postoperative recurrence.

Variable	Clinical/laboratory model HR (95% CI)	Clinical/laboratory model P value	Immune model HR (95% CI)	Immune model P value	Combined model HR (95% CI)	Combined model P value
Pathological TNM stage III vs I–II	4.70 (2.48–8.92)	<0.001	—	—	2.90 (1.39–6.03)	0.004
CEA ≥5 ng/mL	1.49 (0.88–2.51)	0.134	—	—	1.32 (0.77–2.25)	0.313
NLR ≥3.5	2.35 (1.39–3.98)	0.001	—	—	2.98 (1.69–5.25)	<0.001
Albumin, per g/L	0.90 (0.85–0.96)	0.002	—	—	0.91 (0.85–0.97)	0.005
Tregs, per 1% increase	—	—	1.25 (1.11–1.39)	<0.001	1.18 (1.03–1.34)	0.017
MDSCs, per 1% increase	—	—	1.25 (1.05–1.49)	0.012	1.20 (1.00–1.44)	0.045

SE, standard error; HR, hazard ratio; CI, confidence interval; LVI, lymphovascular invasion; CEA, carcinoembryonic antigen; NLR, neutrophil-to-lymphocyte ratio; Treg, regulatory T cell; MDSC, myeloid-derived suppressor cell; NK, natural killer; Alb, albumin.

### 3.6 Kaplan-Meier survival analysis

**Figure 1:**
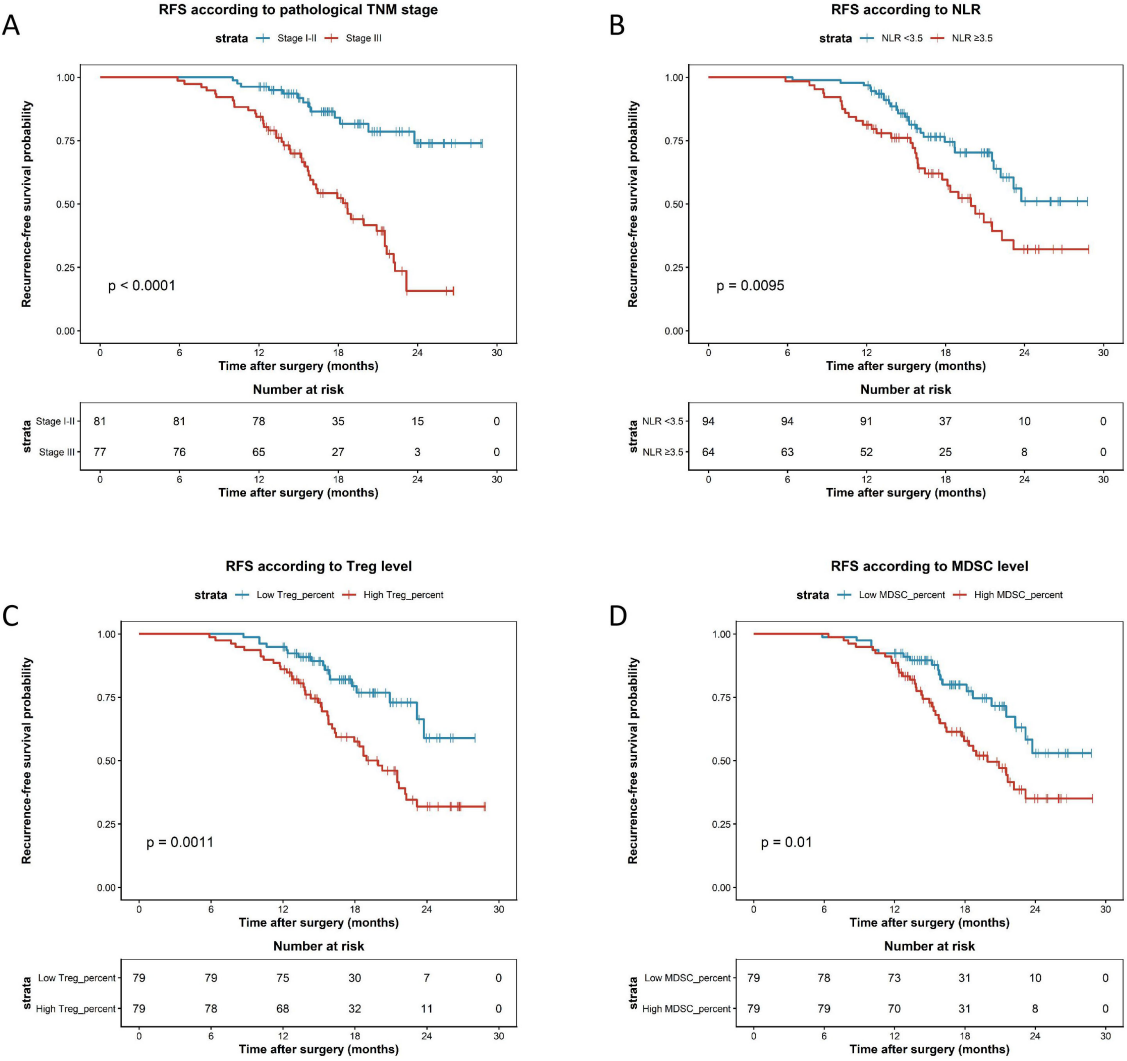
Kaplan-Meier curves for recurrence-free survival according to selected predictors in patients with stage I–III colorectal cancer. Kaplan-Meier curves for recurrence-free survival (RFS) stratified by **(A)** pathological TNM stage, **(B)** neutrophil-to-lymphocyte ratio (NLR), **(C)** Treg level, and **(D)** MDSC level in patients with stage I-III colorectal cancer. TNM stage was categorized as stage I-II versus stage III. NLR was stratified using a cutoff value of 3.5. Treg and MDSC levels were dichotomized according to their median values. P values were calculated using the log-rank test. Risk tables below each plot show the number of patients at risk at each time point.

Kaplan-Meier survival analysis was performed to compare recurrence-free survival (RFS) according to selected clinicopathological, inflammatory, and immune-related variables. Patients with pathological TNM stage III disease showed significantly poorer RFS than those with stage I–II disease (log-rank P < 0.0001). Similarly, patients with elevated NLR (≥3.5) had significantly worse RFS than those with lower NLR levels (log-rank P = 0.0095). When immune cell subsets were analyzed, high Treg levels and high MDSC levels were both associated with significantly reduced RFS compared with their corresponding low-level groups (log-rank P = 0.0011 and P = 0.010, respectively). These findings were consistent with the Cox regression analysis, suggesting that advanced pathological stage, systemic inflammation, and an immunosuppressive peripheral immune profile were associated with increased postoperative recurrence risk (**Figure 1**).

## 4 Discussion

This single-center retrospective cohort study enrolled patients with CRC who underwent curative-intent surgery and had preoperative peripheral blood flow cytometry performed within one week. By including an age- and sex-matched healthy control group, we established a reference framework for evaluating tumor-associated systemic immune alterations. The primary objectives were to characterize systemic immune dysregulation in CRC using peripheral blood immune cell subsets (expressed as percentages of lymphocytes) and to further assess their predictive value for postoperative recurrence risk.

Compared with healthy controls, patients with CRC exhibited decreased proportions of peripheral CD3^+^ T and CD4^+^ T cells, an increased proportion of CD8^+^ T cells, a decreased CD4/CD8 ratio, significantly elevated Tregs and MDSCs, decreased NK cells, and no significant difference in B cells. This pattern suggests a shift in the circulating immune profile toward systemic immune dysregulation. Furthermore, our findings suggest that an increased proportion of CD8^+^ T cells does not necessarily equate to enhanced anti-tumor capacity. Under conditions of persistent antigen stimulation or chronic inflammation, CD8^+^ T cells may undergo functional exhaustion or phenotypic shifts, characterized by reduced effector function, decreased cytotoxic activity, and increased expression of immune checkpoint molecules. This results in an immune state of increased quantity but insufficient effector function, a phenomenon observed in several studies on solid tumors, including CRC (14, 15).

Stratified analysis by disease stage revealed that, compared with patients with stage I-II disease, those with stage III disease had lower proportions of CD4^+^ T cells and NK cells, a higher proportion of CD8^+^ T cells, elevated Tregs and MDSCs, and a decreased CD4/CD8 ratio. These findings suggest that alterations in circulating immune subsets may become more pronounced with advancing pathological stage. However, peripheral blood immune profiles cannot be assumed to directly mirror the tumor immune microenvironment. Paired analyses of circulating immune cells and tumor-infiltrating immune cells are needed to clarify the relationship between systemic and local antitumor immunity in CRC. Taking MDSCs as an example, previous studies have suggested that their peripheral expansion correlates with disease stage and recurrence risk, and their postoperative dynamics may reflect residual tumor or persistent immunosuppression (16). Moreover, research has found that MDSC levels correlate with tumor stage and alterations in T-cell subsets, and their peripheral dynamics may indicate ongoing immunosuppression or residual tumor burden (17). NK cells in CRC often exhibit quantitative and functional impairments, consistent with disease progression (18). This body of evidence, corroborated by the stage-dependent gradients observed in our study, suggests that the peripheral immune profile holds potential value for disease stratification.

In the comparison between the recurrence and non-recurrence groups, patients who developed recurrence had significantly higher Treg and MDSC levels and a lower CD4/CD8 ratio, whereas CD4^+^ T cells, CD8^+^ T cells, and NK cells did not differ significantly between groups. Further multivariate Cox regression analysis identified pathological TNM stage III, NLR ≥3.5, lower albumin level, higher Treg levels, and higher MDSC levels as independent factors associated with shorter RFS. In the combined multivariate Cox model, pathological TNM stage III, NLR ≥3.5, higher Treg levels, and higher MDSC levels were independently associated with an increased risk of recurrence, whereas higher albumin level was associated with a lower recurrence risk. CEA ≥5 ng/mL was not independently associated with recurrence after adjustment. The independent associations of circulating Treg and MDSC levels with shorter RFS suggest that a more immunosuppressive peripheral immune profile may be linked to postoperative recurrence risk. However, because this was an observational study based on peripheral blood measurements, these findings should not be interpreted as evidence of a direct mechanistic role of Tregs or MDSCs in driving recurrence. Rather, elevated circulating Tregs and MDSCs may serve as accessible markers of systemic immune dysregulation associated with unfavorable outcomes. Research indicates that Tregs can promote tumor immune evasion and influence tumor progression by secreting immunosuppressive cytokines and inhibiting effector T-cell responses (19). Concurrently, MDSCs often expand peripherally in patients with CRC and contribute to a sustained immunosuppressive network by suppressing T-cell proliferation, inducing an immunosuppressive microenvironment, and promoting tumor development (16, 20, 21). These findings provide biological context for our observations, but they do not establish that circulating Tregs or MDSCs directly mediate postoperative recurrence in the present cohort.

Although CD4^+^ T-cell proportions were lower in patients with CRC and further decreased in stage III disease, CD4^+^ T cells were not independently associated with postoperative recurrence in the Cox regression analysis. This suggests that reduced peripheral CD4^+^ T-cell levels may reflect disease-related immune alterations rather than serving as an independent predictor of recurrence in the present cohort (22). In contrast, the protective association of albumin underscores the close interplay between nutritional status, systemic inflammation, and host immunity. Hypoalbuminemia may reflect chronic inflammation and tumor-related nutritional depletion, and it may also be linked to impaired immune competence and reduced postoperative recovery capacity, thereby contributing to recurrence risk (23). The independent association between elevated NLR and poorer RFS is consistent with previous evidence. NLR can be regarded as a composite marker of neutrophil-driven inflammation and relatively insufficient lymphocyte-mediated antitumor immunity, and a persistently elevated NLR has been associated with poor prognosis and increased recurrence risk in CRC (10, 24). Notably, pathological TNM stage III remained independently associated with postoperative recurrence in the combined model, confirming the continued prognostic importance of anatomical disease burden. At the same time, the independent effects of NLR, albumin, Tregs, and MDSCs suggest that systemic inflammation, nutritional status, and peripheral immunosuppression provide additional prognostic information beyond pathological stage alone.

Model performance was evaluated using Harrell’s C-index rather than ROC curve analysis, as the present study focused on time-to-event outcomes. Among the three Cox models, the combined model, which incorporated pathological TNM stage, CEA, NLR, albumin, Tregs, and MDSCs, showed the best discriminatory performance, with an apparent C-index of 0.77 and a bootstrap-corrected C-index of 0.75. These findings suggest that postoperative recurrence risk may not be captured by a single immune parameter alone, but rather reflects the combined contribution of pathological disease burden, systemic inflammation, nutritional status, and peripheral immunosuppression. The independent associations of NLR, albumin, Tregs, and MDSCs with recurrence also indicate that host-related inflammatory, nutritional, and immune factors may provide prognostic information beyond pathological stage alone. Similarly, previous studies have highlighted the value of inflammation-related markers in recurrence risk assessment. For instance, Nakamoto et al. (25) found that the systemic immune-inflammation index could predict tumor recurrence after curative resection for CRC, underscoring the important role of systemic inflammation in CRC recurrence. Moreover, NLR, as an indicator reflecting the balance between inflammation and immunity, has been closely associated with survival outcomes and recurrence risk in patients with CRC (26). Together, These findings suggest the potential value of integrating of inflammatory and immune-related markers into recurrence risk assessment models.

This study has several limitations. First, its single-center retrospective design may have introduced selection bias and unmeasured confounding. Second, although recurrence-free survival was analyzed using Kaplan-Meier curves and Cox proportional hazards regression, the relatively limited sample size and number of recurrence events may have restricted the stability of the multivariable models. Third, immune subsets were expressed as percentages of lymphocytes, which may be influenced by fluctuations in absolute lymphocyte counts. Future studies should incorporate absolute immune cell counts and functional immune markers, such as NK-cell cytotoxic molecules or T-cell exhaustion markers, to improve the biological interpretability and robustness of the findings. Fourth, although bootstrap resampling was used for internal validation, external validation in independent cohorts remains necessary. Additional model evaluation approaches, including calibration analysis and decision curve analysis, should also be considered to assess clinical applicability. Another promising direction involves postoperative dynamic immune monitoring. For example, serial assessment of Treg and MDSC trajectories at 1, 3, or 6 months after surgery may facilitate earlier identification of patients at increased risk of recurrence. Future studies may also explore whether peripheral immune profiling can be integrated with minimal residual disease assessment. For example, circulating tumor DNA has been shown to identify molecular residual disease and predict recurrence risk in CRC (27, 28). Combining dynamic immune monitoring with ctDNA or circulating tumor cell detection may provide complementary information on host immune status and residual tumor burden, thereby improving early recurrence risk assessment and individualized follow-up strategies.

## 5 Conclusion

The preoperative peripheral blood immune cell profile reflects systemic immune dysregulation in patients with stage I–III CRC and is associated with postoperative recurrence-free survival. In particular, elevated Treg and MDSC levels, together with pathological TNM stage, NLR, and albumin, may help refine recurrence risk stratification after curative-intent surgery. These findings suggest that peripheral immune profiling may serve as a complementary tool for individualized postoperative follow-up. Prospective multicenter studies with external validation are warranted to confirm its clinical utility.

## Data Availability

The original contributions presented in the study are included in the article/**Supplementary Material**. Further inquiries can be directed to the corresponding author.
