# Supplementary material for: Peripheral blood immune cell subsets predict postoperative recurrence in colorectal cancer: a flow cytometry-based retrospective study
Source: Front Oncol. 2026 Jul 1;16:1846793. doi: 10.3389/fonc.2026.1846793 (PMC13368555; doi:10.3389/fonc.2026.1846793)
Supplement: Supplementary file 1 [file Table1.docx]

Supplementary Table

1. **Discriminative performance and bootstrap internal validation of Cox regression models**

The discriminative performance of the Cox regression models is shown in Supplementary Table 1. The clinical/laboratory model showed an apparent C-index of 0.75, which decreased slightly to 0.73 after bootstrap correction. The immune model had an apparent and bootstrap-corrected C-index of 0.69. The combined model showed the highest discriminative performance, with an apparent C-index of 0.77 and a bootstrap-corrected C-index of 0.75. The mean optimism was low across all models, suggesting limited overfitting after internal validation.

Supplementay Table 1 Discriminative performance and bootstrap internal validation of Cox regression models

| Model | Apparent C-index | Bootstrap-corrected C-index | Mean optimism | Successful bootstrap resamples |
| --- | --- | --- | --- | --- |
| Clinical/laboratory model | 0.75 | 0.73 | 0.01 | 1000 |
| Immune model | 0.69 | 0.69 | 0.01 | 1000 |
| Combined model | 0.77 | 0.75 | 0.02 | 1000 |
